# Supplementary material for: Clinical Evaluation of COVID-19 Survivors at a Public Multidisciplinary Health Clinic
Source: Biomedicines. 2025 Aug 3;13(8):1888. doi: 10.3390/biomedicines13081888 (PMC12383876; doi:10.3390/biomedicines13081888)
Supplement: Supplementary file 1 [file biomedicines-13-01888-s001.zip › Supplemental Material Table S1.pdf]

Supplemental Material Table S1: *Characteristics of the physical examination done in COVID-19 survivors in follow-up according to their final diagnosis (n=113).*

	**Total (n=113)**	**Subacute COVID-19 (n = 41)**	**Post-acute COVID-19 syndrome (n = 72)**	***p***
Anthropometric measurements				
**Weight (kg), mean ± SD**	87.4 ± 18.0	86.6 ± 17.0	87.9 ± 18.6	0.725
**BMI, median (IQR)**	31.3 (16.7 - 47.2)	31.6 (16.7 - 37.7)	31.2 (18.8 - 47.2)	0.703
**BMI categories, n (%)**				0.361
Underweight or normal	10/107 (9.3)	5/38 (13.2)	5/69 (7.2)	
Overweight	30/107 (28.0)	8/38 (21.1)	22/69 (31.9)	
Obese	67/107 (62.6)	25/38 (65.8)	42/69 (60.9)	
**Abdominal circumference (cm), mean ± SD**	103.8 ± 12.9	102.5 ± 14.9	104.1 ± 2.5	0.677
**Altered abdominal circumference according to sex, n (%)**	55/83 (66.3)	10/14 (71.4)	45/69 (62.5)	0.764
**Cervical circumference (cm), mean ± SD**	39.2 ± 3.6	39.7 ± 3.6	39.1 ± 3.6	0.576
Vital signs				
**Heart rate (bpm), mean ± SD**	82.7 ± 14.7	87.4 ± 15.4	80.0 ± 13.8	**0.010**
**MAP in left arm (mmHg), median (IQR)**	96.6 (50.0 - 153.3)	95.8 (71.3 - 153.3)	96.6 (50.0 (129.3)	0.361
**MAP in right arm (mmHg), median, (IQR)**	96.6 (54.0 - 153.3)	93.3 (68.6 - 153.3)	96.6 (54.0 - 133.3)	0.368
**Elevated blood pressure during follow-up, n (%)**	50 (45.0)	15 (38.5)	35 (48.6)	0.325
**Elevated respiratory rate during follow-up, n (%)**	39 (35.8)	19 (50.0)	20 (28.2)	**0.035**
**Oxygen saturation at rest (%), median (IQR)**	96.0 (84.0 - 100.0)	96.0 (85.0 - 100.0)	96.0 (84.0 - 99.0)	0.132
**Reduced oxygen at rest saturation during follow-up, n (%)**	42 (37.5)	18 (43.9)	24 (33.8)	0.316
**Oxygen saturation in exertion (%), median (IQR)**	94.0 (78.0 - 99.0)	94.5 (86.0 - 98.0)	94.0 (78.0 - 99.0)	0.880
**Decreased oxygen saturation at exertion during follow-up, n (%)**	36/59 (61.0)	7/12 (58.3)	29/47 (61.7)	1.000
Neurological examination				
**MINI mental state, n (%)**				0.295
< 7 points	4 (3.6)	0 (0.0)	4 (5.7)	
> = 7 points	107 (96.4)	41 (100.0)	66 (94.3)	
**Total body strength, median (IQR)**	5.0 (4.50 - 5.0)	5.0 (4.6 - 5.0)	5.0 (4.5 - 5.0)	0.532
**Altered gait, n (%)**	14 (12.4)	5 (12.2)	9 (12.5)	1.000
Systematic physical examination				
**Altered oral examination, n (%)**	3 (2.7)	0 (0.0)	3 (4.2)	0.552
**Altered jugular examination, n (%)**	1 (0.9)	0 (0.0)	1 (1.4)	1.000
**Altered cardiovascular examination, n (%)**	6 (5.3)	3 (7.3)	3 (4.2)	0.666
**Altered respiratory examination, n (%)**	17 (15.0)	4 (9.8)	13 (18.1)	0.284
**Altered abdominal examination, n (%)**	14 (12.4)	2 (4.9)	12 (16.7)	0.080
**Altered peripheral pulses on examination, n (%)**	5 (4.4)	0 (0.0)	5 (6.9)	0.157
**Lower limbs oedema, n (%)**	17 (15.0)	4 (9.8)	13 (18.1)	0.284

SD: standard deviation; **IQR:** interquartile range; **BMI:** body mass index; **MAP:** mean arterial pressure.

p values obtained through T-Student test, Fisher test, Chi-Square test, or Mann-Whitney test according to the type and distribution of each variable.
