# Supplementary material for: Clinical Evaluation of COVID-19 Survivors at a Public Multidisciplinary Health Clinic
Source: Biomedicines. 2025 Aug 3;13(8):1888. doi: 10.3390/biomedicines13081888 (PMC12383876; doi:10.3390/biomedicines13081888)
Supplement: Supplementary file 1 [file biomedicines-13-01888-s001.zip › Supplemental Material Table S2.pdf]

**Supplemental Material Table S2:** *Characteristics of the laboratory investigations done in COVID-19 survivors in follow-up according to their final diagnosis (n=113).*

| | Total (n=113) | Subacute COVID-19 (n = 41) | Post-acute COVID-19 syndrome (n = 72) | Missing data, n (%) | p |
|---|---|---|---|---|---|
| **Time from acute onset to laboratory investigations (days), median (IQR)** | 150.0 (117.5 - 197.5) | 125.0 (102.5 - 148.0) | 166.5 (122.5 - 208.7) | 40 (35.4) | **0.009** |
| **Hemoglobin (g/dL), median (IQR)** | 14.1 (13.5 - 15.2) | 14.2 (13.5 - 15.4) | 14.0 (13.4 - 15.2) | 40 (35.4) | 0.715 |
| **Haematocrit (%), median (IQR)** | 41.5 (39.2 - 45.3) | 42.7 (40.0 - 45.5) | 41.4 (35.0 - 62.0) | 40 (35.4) | 0.290 |
| **Lymphocytes (cells/mm³), median (IQR)** | 2450 (1849.5 - 2872.5) | 2124 (1812 - 2724) | 2518 (4888 - 2970) | 40 (35.4) | 0.710 |
| **Leucocytes (cells/mm³), mean ± SD** | 7231.5 ± 2293.6 | 6817.6 ± 2215.6 | 7357.1 ± 2321.5 | 40 (35.4) | 0.399 |
| **Platelets (cells/mm³), median (IQR)** | 241.000 (191.500 - 306.500) | 196.000 (162.000 - 274.500) | 244.000 (202.250 - 307.750) | 40 (35.4) | **0.047** |
| **Eosinophils (cells/mm³), median (IQR)** | 158 (100.5 - 277.0) | 132 (96 - 314) | 162 (100 - 268) | 41 (36.3) | 0.963 |
| **Creatinine (mg/dL), median (IQR)** | 0.8 (0.7 - 1.0) | 0.8 (0.7 - 0.9) | 0.8 (0.72 - 1.01) | 41 (36.3) | 0.730 |
| **Urea (mg/dL), median (IQR)** | 34.0 (30.0 - 43.0) | 33.5 (28.2 - 40.5) | 36.0 (30.5 - 45.3) | 44 (38.9) | 0.319 |
| **Sodium (mg/dL), median (IQR)** | 139.0 (138.0 - 140.5) | 139.5 (138.7 - 141.2) | 139.0 (138.0 - 140.0) | 51 (45.1) | 0.726 |
| **Potassium (mg/dL), median (IQR)** | 4.6 (4.3 - 4.8) | 4.6 (4.2 - 4.8) | 4.6 (4.4 - 4.8) | 43 (38.1) | 0.720 |
| **Serum glucose (mg/dL), median (IQR)** | 98.0 (84.0 - 119.0) | 92.5 (86.7 - 106.7) | 99.0 (90.0 - 122.0) | 42 (37.2) | 0.188 |
| **Hb1Ac (%), median (IQR)** | 5.7 (5.4 - 6.2) | 5.7 (5.3 - 6.0) | 5.7 (5.4 - 6.2) | 58 (51.3) | 0.707 |
| **Serum uric acid (mg/dL), median (IQR)** | 5.8 ± 1.4 | 5.1 ± 0.7 | 5.9 ± 1.5 | 87 (77.0) | 0.249 |
| **Total bilirubin (mg/dL), median (IQR)** | 0.51 (0.40 - 0.68) | 0.51 (0.42 - 0.62) | 0.50 (0.40 - 0.70) | 74 (65.6) | 0.842 |
| **Direct bilirubin (mg/dL), median (IQR)** | 0.08 (0.07 - 0.11) | 0.10 (0.06 - 0.11) | 0.08 (0.70 -0.11) | 73 (64.6) | 0.965 |
| **AST (U/L), median (IQR)** | 20.0 17.0 - 26.0) | 19.0 (16.5 - 22.2) | 20.0 (16.5 - 27.5) | 50 (44.2) | 0.738 |
| **ALT (U/L), median (IQR)** | 21.0 (14.0 - 30.0) | 22.5 (14.7 - 29.0) | 20.5 (14.0 - 30.0) | 51 (45.1) | 0.312 |
| **Alkaline phosphatase (U/L), median (IQR)** | 73.5 (62.2 - 101.5) | 74.0 (59.2 - 98.0) | 73.5 (62.2 - 102.0) | 75 (66.4) | 0.631 |
| **GGT (U/L), median (IQR)** | 31.0 (24.5 - 48.5) | 28.5 (22.0 - 41.0) | 32.0 (24.5 - 55.5) | 76 (67.3) | 0.451 |
| **Fibrinogen (mg/dL), mean ± SD** | 312.2 ± 59.2 | 323.1 ± 62.8 | 308.4 ± 59.4 | 90 (79.6) | 0.611 |
| **TSH (µUI/mL),median (IQR)** | 2.59 (1.69 - 3.62) | 2.59 (1.24 - 4.03) | 2.59 (1.71 - 3.59) | 51 (45.1) | 1.000 |
| **Serum albumin (g/dL), mean ± DP** | 4.33 ± 0.24 | 4.32 ± 0.13 | 4.34 ± 0.27 | 69 (61.1) | 0.792 |
| **Serum ferritin (ng/mL), median (IQR)** | 137.7 (63.8 - 226.6) | 189.6 (62.6 - 339.4) | 132.1 (132.1 - 213.5) | 54 (47.8) | 0.448 |
| **C-reactive protein (mg/dL), median (IQR)** | 4.0 (2.1 - 7.2) | 4.6 (1.1 - 7.1) | 3.7 (2.6 - 9.4) | 77 (68.1) | 0.436 |

**SD:** standard deviation; **IQR:** interquartile range; **Hb1AC:** glycated haemoglobin; **AST:** aspartate aminotransferase; **ALT:** alanine aminotransferase; **GGT:** gamma-glutamyl transferase; **TSH:** thyroid stimulant hormone.
*p* values obtained through T-Student test, Fisher test, Chi-Square test, or Mann-Whitney test according to the type and distribution of each variable.
