# Supplementary material for: Clinical Evaluation of COVID-19 Survivors at a Public Multidisciplinary Health Clinic
Source: Biomedicines. 2025 Aug 3;13(8):1888. doi: 10.3390/biomedicines13081888 (PMC12383876; doi:10.3390/biomedicines13081888)
Supplement: Supplementary file 1 [file biomedicines-13-01888-s001.zip › Supplemental Material Table S3.pdf]

Supplemental Material Table S3: *Characteristics of the physiotherapy follow-up done in COVID-19 survivors according to their final diagnosis (n=56).*

	**Total (n=56)**	**Subacute COVID-19 (n = 22)**	**Post-acute COVID-19 syndrome (n = 34)**	***p***
Inspection, n (%)				
Altered respiratory amplitude	33 (60.0)	16 (72.7)	17 (51.5)	0.162
Altered respiratory expandability	35 (62.5)	17 (77.3)	18 (52.9)	0.092
Altered respiratory elasticity	27 (48.2)	14 (63.6)	13 (38.2)	0.100
Thoracic deformation	1 (1.8)	0 (0.0)	1 (2.9)	1.000
Signs of breathing effort	7 (12.5)	4 (18.2)	3 (8.8)	0.415
Paradoxical respiration	1 (1.8)	1 (4.5)	0 (0.0)	0.393
Intercostal indrawing	2 (3.6)	2 (9.1)	0 (0.0)	0.150
Nose flapping	1 (1.8)	1 (4.5)	0 (0.0)	0.393
Strength tests				
Mean strength of lower limb extension, median (IQR)	4.0 (4.0 - 5.0)	4.0 (4.0 - 4.5)	5.0 (3.5 - 5.0)	0.161
Mean strength of upper limb flexion, median (IQR)	4.0 (4.0 - 5.0)	4.0 (4.0 - 5.0)	5.0 (4.0 - 5.0)	0.149
Right calf circumference, median (IQR)	37.7 (34.2 - 40.0)	36.0 (31.5 - 40.0)	38.0 (36.0 - 40.0)	0.116
Left calf circumference, median (IQR)	38.0 (35.2 - 40.0)	38.0 (35.0 - 39.0)	39.0 (35.5 - 40.0)	0.270
Mean handgrip strength in between sides, median (IQR)	31.7 (27.0 - 40.3)	31.0 (24.0 - 47.5)	32.5 (27.5 - 40.2)	0.617
Manovacuometry				
Peak inspiratory influx, median (IQR)	100.0 (73.7 - 120.0)	80.0 (67.5 - 112.5)	105.0 (76.2 - 120.0)	0.171
% of PiMax reference reached, mean ± SD	102.0 ± 28.7	101.2 ± 40.9	102.7 ± 28.9	0.924
Peak expiratory influx, median (IQR)	80.0 (60.0 - 112.5)	80.0 (55.2 - 110.0)	90.0 (70.0 - 120.0)	0.135
% of PeMax reference reached, mean ± SD	91.9 ± 23.4	75.1 ± 39.3	91.9 ± 17.62	0.172
Peakflow				
Maximum peakflow, mean ± SD	394.6 ± 119.0	408.8 ±139.1	385.2 ± 104.8	0.476
Peakflow reference, mean ± SD	513.4 ± 77.0	505.2 ± 76.4	518.6 ± 79.8	0.683
% of peakflow reference reached, mean ± SD	80.4 ± 23.4	87.6 ± 27.3	74.9 ± 16.4	0.149
6 minute walking test				
Predicted walking distance (PWD), mean ± SD	490.5 ± 39.6	487.4 ± 41.1	492.6 ± 39.1	0.635
Distance walked, mean ± SD	393.8 ± 99.3	440.0 ± 86.0	357.2 ± 95.1	**0.005**
% of PWD walked, mean ± SD	80.3 ± 21.0	89.9 ± 20.2	71.9 ± 18.1	**0.003**
Pre-test oxygen saturation, median (IQR)	95.0 (92.2 - 96.0)	94.5 (92.0 - 96.0)	95.0 (93.0 - 96.7)	0.486
Post-test oxygen saturation (IQR)	93.0 (91.2 - 95.7)	93.0 (91.5 - 95.0)	94.0 (90.5 - 96.0)	0.983
Pre-test heart rate, median (IQR)	86.0 (78.2 - 102.2)	88.0 (80.5 - 103.7)	85.0 (71.2 - 100.0)	0.341
Post-test heart rate, median (IQR)	94.5 (85.0 - 108.2)	89.0 (85.0 - 103.0)	95.5 (85.0 - 109.7)	0.579
Diference between pre and post-test BORG test, median (IQR)	2.0 (1.0 - 3.0)	1.0 (0.0 - 2.0)	2.0 (1.0 - 3.0)	0.149

SD: standard deviation; **IQR:** interquartile range; **PiMax:** peak inspiratory influx; **PeMax:** peak expiratory influx; **PWD:** predicted walking distance.
p values obtained through T-Student test, Fisher test, Chi-Square test, or Mann-Whitney test according to the type and distribution of each variable.
