# Supplementary material for: Clinical Evaluation of COVID-19 Survivors at a Public Multidisciplinary Health Clinic
Source: Biomedicines. 2025 Aug 3;13(8):1888. doi: 10.3390/biomedicines13081888 (PMC12383876; doi:10.3390/biomedicines13081888)
Supplement: Supplementary file 1 [file biomedicines-13-01888-s001.zip › Supplemental Material Table S4.pdf]

Supplemental Material Table S4: *Binary logistic regression with diagnosis of post-acute COVID-19 syndrome as dependent variable. .*

	Model 0				Model 1				Model 2			
	β	*OR*	*CI 95%*	*p*	*β*	*OR*	*CI 95%*	*p*	*β*	*OR*	*CI 95%*	*p*
**Constant**	0.556	1.744		**0.006**	-5.068	0.006		**<0.001**	-5.135	0.006		**<0.001**
**Days from acute onset to follow-up**					0.058	1.060	(1.035–1.084)	**<0.001**	0.058	1.060	(1.035–1.084)	**<0.001**
**Male sex**									0.111	1.117	(0.221-5.640)	0.893
**Number of previous hospitalizations**												
**Number of comorbidities**												
**Complete COVID-19 vaccination**												
**Length of hospital stay**												

	Model 3				Model 4				Model 5			
	β	*OR*	*CI 95%*	*p*	*β*	*OR*	*CI 95%*	*p*	*β*	*OR*	*CI 95%*	*p*
**Constant**	-4.742	0.009		**<0.001**	-4.875	0.008		**<0.001**	-4.761	0.009		**0.001**
**Days from acute onset to follow-up**	0.058	1.060	(1.036-1.085)	**<0.001**	0.057	1.059	(1.033-1.085)	**<0.001**	0.057	1.058	(1.033-1.084)	**<0.001**
**Male sex**	-0.168	0.846	(0.150-4.770)	0.846	-0.602	0.548	(0.087-3.437)	0.521	-0.573	0.564	(0.087-3.641)	0.547
**Number of previous hospitalizations**	-0.349	0.705	(0.332-1.497)	0.363	-0.598	0.550	(0.227-1.335)	0.186	-0.565	0.569	(0.215-1.504)	0.255
**Number of comorbidities**	0.018	1.018	(0.760-1.364)	0.903	0.054	1.056	(0.786-1.418)	0.720	0.050	1.051	(0.779-1.419)	0.745
**Complete COVID-19 vaccination**					1.554	4.730	(0.704-31.781)	0.110	1.551	4.717	(0.700-31.77)	0.111
**Length of hospital stay**									-0.009	0.991	(0.897-1.096)	0.866

OR: odds ratio; **95% CI:** 95% confidence interval.
