# Supplementary material for: Clinical Evaluation of COVID-19 Survivors at a Public Multidisciplinary Health Clinic
Source: Biomedicines. 2025 Aug 3;13(8):1888. doi: 10.3390/biomedicines13081888 (PMC12383876; doi:10.3390/biomedicines13081888)
Supplement: Supplementary file 1 [file biomedicines-13-01888-s001.zip › TCLE English (translated version) .pdf]

INFORMED CONSENT FORM – ADULT OVER 18 YEARS OLD / UNABLE TO CONSENT
HOSPITAL UNIVERSITÁRIO CAJURU

I have been invited as a volunteer to participate in the study titled *"Clinical and epidemiological characterization of patients treated in Curitiba, PR,"* which aims to describe the progression of suspected and confirmed hospitalized COVID-19 cases in Curitiba during the 2020 pandemic. We believe this study is important because information about the evolution of suspected cases of the disease has great clinical and scientific value, supporting decision-making and improving understanding of this new illness that has affected the entire world.

PARTICIPATION IN THE STUDY
My participation in the study will consist of allowing observation and analysis of my medical records, as well as potentially undergoing imaging or laboratory tests such as complete blood count, cellular profile analysis (blood collection), inflammatory biomarker dosage (serum collection), echocardiograms (heart ultrasound), and echocardiography, always prioritizing the patient's well-being.

RISKS AND BENEFITS
I was informed that I may expect some benefits from participating in this study, including indirect benefits such as contributing to the understanding of the pandemic and helping address potential future epidemics. I was also informed that the following discomforts or risks may occur: the risks inherent to the patient's routine treatment, i.e., the same to which the patient would be exposed whether or not participating in the study. These may include phlebitis or bruising associated with mild discomfort at the blood collection site. Measures will be taken to reduce such risks, including strict adherence to institutional protocols for each procedure and ensuring that all collections are performed by trained personnel.

CONFIDENTIALITY AND PRIVACY
I am aware that my privacy will be respected, meaning that my name or any other data or element that could in any way identify me will be kept confidential. The researchers are responsible for safeguarding and maintaining the confidentiality of all data and ensuring that no research information is publicly disclosed.

AUTONOMY
Assistance is guaranteed throughout the research. I am also assured full access to all information and clarifications regarding the study and its consequences—everything I wish to know before, during, and after my participation. I was also informed that I may refuse to participate in the study or withdraw my consent at any time without needing to justify my decision, and that this will not affect the care I receive.

REIMBURSEMENT AND COMPENSATION
All expenses will be covered by the researchers. Research participants and their companions (if any) will not incur any costs related to procedures, tests, transportation, food, or other expenses. If any costs are incurred, reimbursement will be provided as follows: bank deposit. Likewise, if any harm results from my participation in the study, I will receive appropriate compensation.

INITIALS OF THE RESEARCH PARTICIPANT

INITIALS OF THE RESEARCHER

CONTACT

The researchers involved in the study are Juliano Gasparetto, Cristina Baena, Anna Flavia Miggiolaro, Felipe Tuon, Andrea Moreno, Lucia de Noronha, Thyago Moraes, Lidia Moura, José Faria, and Gustavo Lenci. They are researchers and professors at the PUCPR School of Medicine and can be contacted at (41) 3271-2979.

The National Research Ethics Committee (CONEP) is a group responsible for ensuring that your rights as a research participant are respected. It is responsible for assessing whether the research has been planned and conducted ethically. If you believe that the research is not being conducted as expected or if you feel harmed in any way, you may contact CONEP by phone at +55 (61) 3315-5877 (Monday to Friday, 8 a.m. to 6 p.m.) or by email at conep@saude.gov.br Address: SRTVN 701, Via W5 Norte, Lot D, 3rd Floor, Brasília – Brazil.

DECLARATION

**Research participant's information**	
Name:	
Phone:	
e-mail:	

**Responsible guardian's information**	
Name:	
Phone:	
Relationship:	

Curitiba, _____ de ___________________ de _________

____
Signature of the research participant

____
Signature of the researcher

INITIALS OF THE RESEARCH PARTICIPANT

INITIALS OF THE RESEARCHER
