# Supplementary material for: Clinical Evaluation of COVID-19 Survivors at a Public Multidisciplinary Health Clinic
Source: Biomedicines. 2025 Aug 3;13(8):1888. doi: 10.3390/biomedicines13081888 (PMC12383876; doi:10.3390/biomedicines13081888)
Supplement: Supplementary file 1 [file biomedicines-13-01888-s001.zip › TCLE Português (versão original).pdf]

TERMO DE CONSENTIMENTO LIVRE E ESCLARECIDO – MAIOR DE 18 ANOS / INCAPAZ DE CONSENTIR
HOSPITAL UNIVERSITÁRIO CAJURU

Estou sendo convidado(a) como voluntário(a) a participar do estudo "Caracterização clínica e epidemiológica de pacientes atendidos em Curitiba PR" e que tem como objetivo descrever a evolução dos casos internados suspeitos e confirmados de COVID-19 em Curitiba durante a pandemia de 2020. Acreditamos que ela seja importante porque informações sobre a evolução dos casos suspeitos da doença são de grande valor clínico e científico, ajudando a tomada de decisão e compreensão dessa nova doença que tem afetado o mundo inteiro.

PARTICIPAÇÃO NO ESTUDO

Minha participação no estudo será de permitir a observação e análise de meu prontuário médico assim como eventualmente me submeter a exames de imagem ou laboratoriais como hemogramas (exame de sangue), análise do perfil celular (coleta de sangue), dosagem de biomarcadores inflamatório (coleta de soro), ecocardiogramas (ultrassom do coração), ecocardiografia, prezando sempre pelo bem-estar do paciente.

RISCOS E BENEFÍCIOS

Fui alertado de que, da pesquisa a se realizar, posso esperar alguns benefícios, tais como benefícios indiretos de contribuição para a compreensão da pandemia e o enfrentamento de possíveis novas epidemias. Recebi, também que é possível que aconteçam os seguintes desconfortos ou riscos como os riscos inerentes ao tratamento de rotina do paciente, ou seja os mesmos aos quais o paciente se submete durante o seu tratamento participando ou não do estudo, dentre os quais temos flebite, hematomas associados a leve desconforto no local da coleta do hemograma. Dos quais medidas serão tomadas para sua redução, tais como o cumprimento dos protocolos hospitalares instituídos para a execução de cada exame, a coleta dos exames ser feita por pessoal capacitado.

SIGILO E PRIVACIDADE

Estou ciente de minha privacidade respeitada, ou seja, meu nome ou qualquer outro dado ou elemento que possa, de qualquer forma me identificar será mantido em sigilo. Os pesquisadores se responsabilizam pela guarda e confidencialidade dos dados, bem como a não exposição dos dados de pesquisa.

AUTONOMIA

É assegurada a assistência durante toda pesquisa, bem como me é garantido o livre acesso a todas as informações e esclarecimentos adicionais sobre o estudo e suas consequências, enfim, tudo que eu queira saber antes, durante e depois da minha participação. Também fui informado de que posso recusar a participar no estudo, ou retirar o consentimento a qualquer momento, sem precisar justificar, e de, por desejar sair da pesquisa, este não sofrerá qualquer prejuízo à assistência que vem sendo recebida.

RÚBRICA DO SUJEITO DE PESQUISA

RÚBRICA DO PESQUISADOR

RESSARCIMENTO E INDENIZAÇÃO

Todas as despesas serão de responsabilidade dos Pesquisadores. Os participantes da pesquisa bem como seu acompanhante (se houver) não arcarão com nenhum custo referente a procedimento, exames, transporte, alimentação ou outros. Caso ocorra qualquer despesa, haverá ressarcimento dos valores gastos na forma seguinte: depósito em conta. De igual maneira, caso ocorra algum dano decorrente da minha participação no estudo, serei devidamente indenizado.

CONTATO

Os pesquisadores envolvidos com o estudo são Juliano Gasparetto, Cristina Baena, Anna Flavia Miggiolaro, Felipe Tuon, Andrea Moreno, Lucia de Noronha, Thyago Moraes, Lidia Moura, José Faria, Gustavo Lenci, que são pesquisadores e professores na Escola de Medicina da PUCPR e com eles poderei manter contato pelos telefones (41)3271-2979.

O Conselho Nacional de Pesquisa (CONEP) é composto por um grupo de pessoas que estão trabalhando para garantir que seus direitos como participante de pesquisa sejam respeitados. Ele tem a obrigação de avaliar se a pesquisa foi planejada e se está sendo executada de forma ética. Se você achar que a pesquisa não está sendo realizada da forma como você imaginou ou que está sendo prejudicado de alguma forma, você pode entrar em contato com o CONEP pelo telefone (61)3315-5877 entre segunda e sexta-feira das 08h às 18h ou pelo e-mail conep@saude.gov.br Endereço: SRTVN 701, Via W 5 Norte, Lote D 3º andar Brasilia.

DECLARAÇÃO

**Dados do participante da pesquisa**	
Nome:	
Telefone:	
e-mail:	

**Dados do responsável pelo participante da pesquisa**	
Nome:	
Telefone:	
Grau de parentesco:	

Curitiba, _____ de ___________________ de _________

_______________________________________ _______________________________________

Assinatura do participante da pesquisa Assinatura do pesquisador

RÚBRICA DO SUJEITO DE PESQUISA

RÚBRICA DO PESQUISADOR
